# (*E*)-Benz­yl(4-{[1-(prop-2-en-1-yl)-1*H*-1,2,3-triazol-4-yl]meth­oxy}benzyl­idene)amine

**DOI:** 10.1107/S1600536814002645

**Published:** 2014-02-08

**Authors:** Mehmet Akkurt, Aliasghar Jarrahpour, Mehdi Mohammadi Chermahini, Pezhman Shiri, Namık Özdemir

**Affiliations:** a Department of Physics, Faculty of Sciences, Erciyes University, 38039 Kayseri, Turkey; b Department of Chemistry, College of Sciences, Shiraz University, 71454 Shiraz, Iran; c Department of Physics, Faculty of Arts and Sciences, Ondokuz Mayıs University, 55139 Samsun, Turkey

## Abstract

The triazole ring of the title compound, C_20_H_20_N_4_O, is normal to the central benzene ring, making a dihedral angle of 90.0 (3)°, and forms a dihedral angle of 69.2 (3)° with the terminal phenyl ring. The dihedral angle between the phenyl and benzene rings is 88.2 (3)°. The atoms of the terminal propenyl group are disordered over two sets of sites, with a site-occupancy ratio of 0.663 (13):0.337 (13). In the crystal, C—H⋯N contacts lead to the formation of a layer structure extending parallel to (011). Two weak C—H⋯π inter­actions are also observed.

## Related literature   

For background to the importance of Schiff bases and triazole derivatives and their uses, see: Calligaris & Randaccio (1987 ▶); Dikusar & Kozlov (2006 ▶); Macho *et al.* (2004 ▶); Yap & Weinreb (2006 ▶); Yu *et al.* (2006 ▶). For similar structures, see: Akkurt *et al.* (2013*a* ▶,*b* ▶).
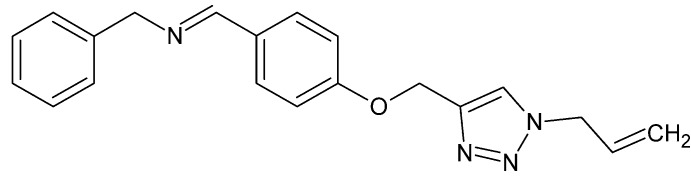


## Experimental   

### 

#### Crystal data  

C_20_H_20_N_4_O
*M* *_r_* = 332.40
Monoclinic, 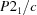

*a* = 8.5873 (9) Å
*b* = 20.0601 (13) Å
*c* = 10.7450 (9) Å
β = 97.241 (8)°
*V* = 1836.2 (3) Å^3^
*Z* = 4
Mo *K*α radiation
μ = 0.08 mm^−1^
*T* = 296 K
0.57 × 0.24 × 0.05 mm

#### Data collection  

Stoe IPDS 2 diffractometer
Absorption correction: integration (*X-RED32*; Stoe & Cie, 2002 ▶) *T*_min_ = 0.971, *T*_max_ = 0.996
10252 measured reflections
3236 independent reflections
981 reflections with *I* > 2σ(*I*)
*R*_int_ = 0.137

#### Refinement  

*R*[*F* ^2^ > 2σ(*F* ^2^)] = 0.079
*wR*(*F* ^2^) = 0.117
*S* = 0.93
3236 reflections
236 parameters
6 restraints
H-atom parameters constrained
Δρ_max_ = 0.16 e Å^−3^
Δρ_min_ = −0.11 e Å^−3^

### 

Data collection: *X-AREA* (Stoe & Cie, 2002 ▶); cell refinement: *X-AREA*; data reduction: *X-RED32* (Stoe & Cie, 2002 ▶); program(s) used to solve structure: *SHELXS2013* (Sheldrick, 2008 ▶); program(s) used to refine structure: *SHELXL2013* (Sheldrick, 2008 ▶); molecular graphics: *ORTEP-3 for Windows* (Farrugia, 2012 ▶); software used to prepare material for publication: *WinGX* (Farrugia, 2012 ▶).

## Supplementary Material

Crystal structure: contains datablock(s) global, I. DOI: 10.1107/S1600536814002645/sj5391sup1.cif

Structure factors: contains datablock(s) I. DOI: 10.1107/S1600536814002645/sj5391Isup2.hkl

Supporting information file. DOI: 10.1107/S1600536814002645/sj5391Isup3.cml

CCDC reference:

Additional supporting information:  crystallographic information; 3D view; checkCIF report

## Figures and Tables

**Table 1:** Hydrogen-bond geometry (Å, °) *Cg*2 is the centroid of the C1–C6 phenyl ring.

*D*—H⋯*A*	*D*—H	H⋯*A*	*D*⋯*A*	*D*—H⋯*A*
C17—H17⋯N3^i^	0.93	2.54	3.379 (7)	150
C18*B*—H18*D*⋯N1^ii^	0.97	2.52	3.42 (2)	153
C13—H13⋯*Cg*2^iii^	0.93	2.88	3.638 (6)	139
C18*B*—H18*C*⋯*Cg*2^iv^	0.97	2.95	3.706 (18)	135

Symmetry codes: (i) 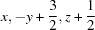; (ii) 
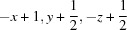
; (iii) 
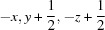
; (iv) 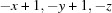.

**Fig. 1.**
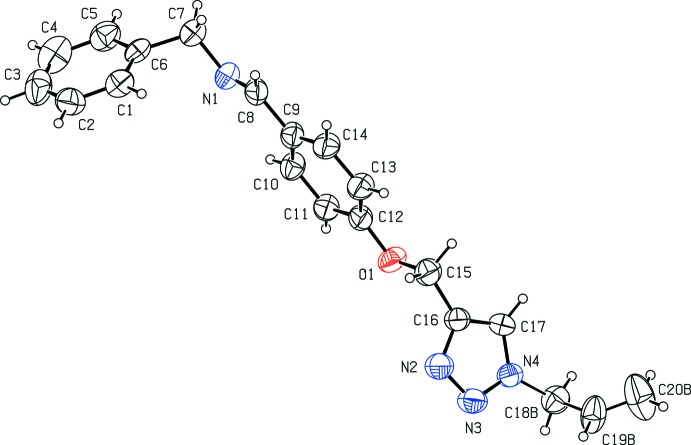
View of the title molecule (I) with the atom numbering scheme. Displacement ellipsoids for non-H atoms are drawn at the 30% probability level. Only major disorder component is shown.

**Fig. 2.**
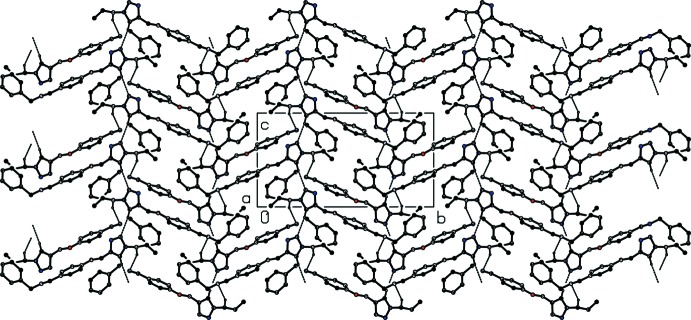
Hydrogen bonding and molecular packing of (I) viewed along the a axis. Only H atoms involved in H bonding and atoms of the major disorder component are shown.

**Fig. 3.**
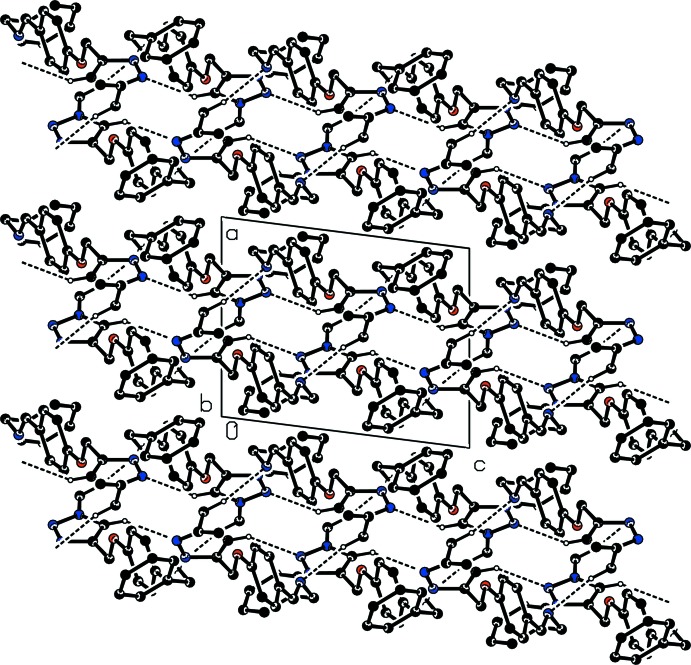
Hydrogen bonding and molecular packing of (I) viewed along the b axis. Only H atoms involved in H bonding and atoms of the major disorder component are shown.

## supplementary crystallographic information

### 1. Comment

Schiff bases result from the condensation of primary amines with carbonyl compounds to give imines containing a C=N bond (Calligaris & Randaccio 1987). There is a continuing interest in the chemistry of Schiff bases and their complexes because of their uses as biologically active substances, liquid crystals, dyes, luminophores and polymer stabilizers (Dikusar & Kozlov 2006). Schiff bases are used as substrates in the preparation of a large number of bioactive and industrial compounds *via* ring closure, cycloaddition, replacement reactions, cyclization and enantioselective oxidation (Macho *et al.*, 2004). 1,2,3-Triazoles are nitrogen heterocycles, that have a number of important industrial, agrochemical, and pharmaceutical uses (Yap & Weinreb 2006). Triazole derivatives also display a broad range of biological activity, showing potential applications as antitumor, antibacterial, antifungal and antiviral agents (Yu *et al.*, 2006). Therefore, compound (I), which that contains both of these features, was synthesized and its X-ray structure is reported here.

In the title compound (I, Fig. 1), the C1–C6 phenyl and C9–C14 benzene rings make a dihedral angle of 88.2 (3)° with each other. The plane of the five-membered triazole ring (N2–N4/C16/C17) of (I) lies perpendicular to the plane of the central benzene ring (C9–C14) with a dihedral angle of 90.0 (3)° and, forms dihedral angles of 69.2 (3)°, with the C1–C6 phenyl ring.

The C6–C7–N1–C8, C12–O1–C15–C16, N4–C18A–C19A–C20A and N4–C18B–C19B–C20B torsion angles are 179.7 (4), -174.2 (3), -174.2 (3) and 151.9 (14)°, respectively. The values of the bond lengths and bond angles in (I) are comparable to those reported for the similar compounds (Akkurt *et al.*, 2013*a*,*b*).

In the crystal structure, intermolecular C—H···N contacts (Table 1; Figs. 2 & 3) connect the adjacent molecules, forming a layer structure extending parallel to the (011) plane. In addition, weak two C—H···π interactions (Table 1) contribute to the stabilization of the molecular packing.

### 2. Experimental

Reaction of 4-((1-allyl-1*H*-1,2,3-triazol-4-yl)methoxy)benzaldehyde (1.00 mmol) with phenylmethanamine (1.00 mmol) in refluxing ethanol gave the title compound (I). Recrystallization from ethanol gave colourless prisms in 75% yield. Mp: 373–375 K. IR (KBr, cm^-1^):1635 (C=N). ^1^H-NMR (250 MHz, CDCl_3_), δ (p.p.m.): 4.78 (CH_2_, s, 2H), 4.96 (d, 2H, J=5 Hz), 5.24 (s, 2H), 5.35 (m, 2H), 6.01 (m, 1H), 7.01 (aromatic H, d, 2H, J=7.5 Hz), 6.24–7.33 (aromatic H, m, 5H), 7.72 (aromatic H, d, 2H, J=10 Hz), 7.61 (H triazole, s, 1H), 8.32 (HC═N, s, 1H). ^13^CNMR (62.9 MHz, CDCl_3_), δ (p.p.m): 52.7, 60.0 (CH_2_—N), 64.9 (CH_2_—O), 114.7–143.9 (aromatic carbons and C=C triazole), 161.1 (C=N).

### 3. Refinement

All H atoms were positioned geometrically and were refined using a riding model with *U*_iso_(H) = 1.2*U*_eq_(C). The atoms of the propenyl group are disordered over two positions with a site-occupancy ratio of 0.663 (13): 0.337 (13). The small proportion of reflections observed is a result of the rather poor quality of the very thin crystals obtained.

### Crystal data

**Table:**

C_20_H_20_N_4_O	*F*(000) = 704
*M**_r_* = 332.40	*D*_x_ = 1.202 Mg m^−^^3^
Monoclinic, *P*2_1_/*c*	Mo *K*α radiation, λ = 0.71073 Å
Hall symbol: -P 2ybc	Cell parameters from 4967 reflections
*a* = 8.5873 (9) Å	θ = 1.9–27.9°
*b* = 20.0601 (13) Å	µ = 0.08 mm^−^^1^
*c* = 10.7450 (9) Å	*T* = 296 K
β = 97.241 (8)°	Prism, colourless
*V* = 1836.2 (3) Å^3^	0.57 × 0.24 × 0.05 mm
*Z* = 4	

### Data collection

**Table:**

Stoe IPDS 2 diffractometer	3236 independent reflections
Radiation source: sealed X-ray tube, 12 x 0.4 mm long-fine focus	981 reflections with *I* > 2σ(*I*)
Plane graphite monochromator	*R*_int_ = 0.137
Detector resolution: 6.67 pixels mm^-1^	θ_max_ = 25.0°, θ_min_ = 2.0°
ω scans	*h* = −9→10
Absorption correction: integration (*X-RED32*; Stoe & Cie, 2002)	*k* = −23→23
*T*_min_ = 0.971, *T*_max_ = 0.996	*l* = −12→12
10252 measured reflections	

### Refinement

**Table:**

Refinement on *F*^2^	6 restraints
Least-squares matrix: full	Hydrogen site location: inferred from neighbouring sites
*R*[*F*^2^ > 2σ(*F*^2^)] = 0.079	H-atom parameters constrained
*wR*(*F*^2^) = 0.117	*w* = 1/[σ^2^(*F*_o_^2^) + (0.0181*P*)^2^] where *P* = (*F*_o_^2^ + 2*F*_c_^2^)/3
*S* = 0.93	(Δ/σ)_max_ < 0.001
3236 reflections	Δρ_max_ = 0.16 e Å^−^^3^
236 parameters	Δρ_min_ = −0.11 e Å^−^^3^

### Special details

**Table:**

Geometry. Bond distances, angles *etc*. have been calculated using the rounded fractional coordinates. All su's are estimated from the variances of the (full) variance-covariance matrix. The cell e.s.d.'s are taken into account in the estimation of distances, angles and torsion angles
Refinement. Refinement on *F*^2^ for ALL reflections except those flagged by the user for potential systematic errors. Weighted *R*-factors *wR* and all goodnesses of fit *S* are based on *F*^2^, conventional *R*-factors *R* are based on *F*, with *F* set to zero for negative *F*^2^. The observed criterion of *F*^2^ > σ(*F*^2^) is used only for calculating -*R*-factor-obs *etc*. and is not relevant to the choice of reflections for refinement. *R*-factors based on *F*^2^ are statistically about twice as large as those based on *F*, and *R*-factors based on ALL data will be even larger.

### Fractional atomic coordinates and isotropic or equivalent isotropic displacement parameters (Å^2^)

**Table:**

	*x*	*y*	*z*	*U*_iso_*/*U*_eq_	Occ. (<1)
O1	0.3201 (4)	0.56064 (18)	0.0649 (3)	0.0860 (16)	
N1	0.2214 (5)	0.2770 (2)	0.3186 (4)	0.089 (2)	
N2	0.2721 (5)	0.6732 (2)	−0.1458 (5)	0.091 (2)	
N3	0.3496 (6)	0.7255 (3)	−0.1800 (4)	0.092 (2)	
N4	0.4179 (5)	0.7540 (2)	−0.0749 (5)	0.0762 (17)	
C1	0.0466 (7)	0.1614 (3)	0.1724 (7)	0.092 (3)	
C2	0.0451 (9)	0.1104 (4)	0.0881 (6)	0.114 (3)	
C3	0.1458 (10)	0.0586 (4)	0.1125 (9)	0.125 (4)	
C4	0.2454 (8)	0.0562 (4)	0.2212 (9)	0.127 (4)	
C5	0.2463 (8)	0.1079 (4)	0.3075 (6)	0.104 (3)	
C6	0.1454 (7)	0.1609 (3)	0.2823 (6)	0.075 (3)	
C7	0.1495 (7)	0.2187 (3)	0.3719 (5)	0.099 (3)	
C8	0.1315 (6)	0.3256 (3)	0.2899 (5)	0.082 (3)	
C9	0.1839 (7)	0.3873 (3)	0.2328 (5)	0.075 (2)	
C10	0.3292 (6)	0.3939 (3)	0.1924 (5)	0.079 (2)	
C11	0.3695 (6)	0.4523 (3)	0.1362 (5)	0.077 (2)	
C12	0.2673 (6)	0.5053 (3)	0.1221 (5)	0.074 (2)	
C13	0.1217 (6)	0.4994 (3)	0.1635 (5)	0.084 (3)	
C14	0.0817 (6)	0.4405 (3)	0.2180 (5)	0.084 (2)	
C15	0.2156 (6)	0.6170 (3)	0.0470 (5)	0.090 (3)	
C16	0.2921 (6)	0.6707 (3)	−0.0195 (6)	0.074 (2)	
C17	0.3832 (6)	0.7217 (3)	0.0260 (5)	0.082 (3)	
C18B	0.512 (2)	0.8153 (7)	−0.072 (2)	0.090 (4)	0.663 (13)
C19B	0.4081 (17)	0.8751 (6)	−0.0933 (16)	0.112 (6)	0.663 (13)
C20B	0.415 (4)	0.9226 (11)	−0.0118 (19)	0.174 (9)	0.663 (13)
C20A	0.382 (8)	0.918 (2)	−0.077 (4)	0.174 (9)	0.337 (13)
C18A	0.503 (6)	0.8150 (14)	−0.104 (4)	0.090 (4)	0.337 (13)
C19A	0.458 (3)	0.8683 (12)	−0.015 (3)	0.112 (6)	0.337 (13)
H2	−0.02440	0.11120	0.01440	0.1370*	
H5	0.31470	0.10670	0.38190	0.1250*	
H3	0.14660	0.02430	0.05430	0.1500*	
H4	0.31270	0.02010	0.23780	0.1530*	
H1	−0.02120	0.19720	0.15450	0.1100*	
H10	0.40050	0.35880	0.20280	0.0950*	
H11	0.46700	0.45590	0.10770	0.0930*	
H13	0.05130	0.53490	0.15470	0.1010*	
H14	−0.01650	0.43660	0.24520	0.1010*	
H15A	0.19210	0.63320	0.12760	0.1080*	
H15B	0.11790	0.60380	−0.00210	0.1080*	
H17	0.41500	0.73210	0.10960	0.0990*	
H18C	0.58140	0.81320	−0.13650	0.1080*	0.663 (13)
H18D	0.57620	0.81910	0.00870	0.1080*	0.663 (13)
H19B	0.33780	0.87810	−0.16630	0.1340*	0.663 (13)
H20C	0.48460	0.92000	0.06150	0.2100*	0.663 (13)
H20D	0.34920	0.95950	−0.02640	0.2100*	0.663 (13)
H7A	0.04360	0.22950	0.38750	0.1180*	
H7B	0.20970	0.20660	0.45120	0.1180*	
H8	0.02760	0.32260	0.30530	0.0980*	
H18A	0.47240	0.82830	−0.19040	0.1080*	0.337 (13)
H18B	0.61520	0.80750	−0.09120	0.1080*	0.337 (13)
H19A	0.48070	0.86620	0.07150	0.1340*	0.337 (13)
H20A	0.36230	0.91730	−0.16370	0.2100*	0.337 (13)
H20B	0.34760	0.95410	−0.03250	0.2100*	0.337 (13)

### Atomic displacement parameters (Å^2^)

**Table:**

	*U*^11^	*U*^22^	*U*^33^	*U*^12^	*U*^13^	*U*^23^
O1	0.087 (3)	0.073 (2)	0.105 (3)	0.010 (2)	0.040 (2)	0.020 (2)
N1	0.108 (4)	0.069 (3)	0.091 (4)	−0.020 (3)	0.019 (3)	−0.001 (3)
N2	0.094 (4)	0.098 (4)	0.079 (4)	−0.014 (3)	0.009 (3)	0.004 (3)
N3	0.098 (4)	0.106 (4)	0.071 (3)	−0.013 (3)	0.009 (3)	0.008 (3)
N4	0.083 (3)	0.076 (3)	0.070 (3)	−0.007 (3)	0.011 (3)	0.007 (3)
C1	0.089 (4)	0.087 (5)	0.097 (5)	−0.006 (4)	0.006 (4)	0.011 (4)
C2	0.140 (7)	0.118 (6)	0.089 (5)	−0.032 (5)	0.029 (4)	−0.007 (5)
C3	0.119 (7)	0.089 (6)	0.182 (9)	−0.020 (5)	0.074 (6)	−0.027 (6)
C4	0.082 (6)	0.111 (7)	0.188 (9)	0.005 (5)	0.018 (5)	0.016 (7)
C5	0.097 (5)	0.101 (5)	0.113 (6)	−0.015 (5)	0.005 (4)	0.011 (5)
C6	0.071 (4)	0.065 (4)	0.090 (5)	−0.014 (3)	0.018 (4)	0.016 (4)
C7	0.128 (5)	0.076 (4)	0.095 (5)	−0.019 (4)	0.023 (4)	0.004 (4)
C8	0.092 (5)	0.069 (4)	0.088 (4)	−0.014 (4)	0.025 (4)	−0.014 (4)
C9	0.077 (4)	0.065 (4)	0.084 (4)	−0.008 (3)	0.015 (3)	−0.008 (4)
C10	0.073 (4)	0.068 (4)	0.098 (4)	0.002 (3)	0.015 (3)	0.000 (4)
C11	0.066 (4)	0.077 (4)	0.093 (4)	0.001 (3)	0.030 (3)	−0.005 (4)
C12	0.077 (4)	0.068 (4)	0.082 (4)	0.002 (3)	0.028 (3)	−0.006 (3)
C13	0.079 (4)	0.079 (4)	0.100 (5)	0.003 (3)	0.034 (3)	0.003 (4)
C14	0.078 (4)	0.082 (4)	0.100 (4)	−0.008 (4)	0.039 (3)	0.001 (4)
C15	0.099 (5)	0.078 (4)	0.099 (5)	0.009 (4)	0.032 (3)	0.003 (4)
C16	0.081 (4)	0.074 (4)	0.068 (4)	0.003 (3)	0.017 (3)	0.007 (4)
C17	0.108 (5)	0.084 (4)	0.058 (4)	−0.003 (4)	0.024 (3)	0.002 (4)
C18B	0.080 (6)	0.091 (5)	0.099 (11)	−0.004 (4)	0.016 (7)	0.003 (6)
C19B	0.112 (10)	0.072 (6)	0.137 (14)	0.017 (6)	−0.040 (8)	−0.008 (9)
C20B	0.177 (17)	0.156 (10)	0.18 (2)	0.048 (10)	−0.012 (17)	−0.071 (14)
C20A	0.177 (17)	0.156 (10)	0.18 (2)	0.048 (10)	−0.012 (17)	−0.071 (14)
C18A	0.080 (6)	0.091 (5)	0.099 (11)	−0.004 (4)	0.016 (7)	0.003 (6)
C19A	0.112 (10)	0.072 (6)	0.137 (14)	0.017 (6)	−0.040 (8)	−0.008 (9)

### Geometric parameters (Å, º)

**Table:**

O1—C12	1.373 (7)	C19A—C20A	1.32 (5)
O1—C15	1.441 (7)	C19B—C20B	1.29 (3)
N1—C7	1.472 (7)	C1—H1	0.9300
N1—C8	1.258 (7)	C2—H2	0.9300
N2—N3	1.319 (7)	C3—H3	0.9300
N2—C16	1.347 (8)	C4—H4	0.9300
N3—N4	1.334 (7)	C5—H5	0.9300
N4—C17	1.329 (7)	C7—H7A	0.9700
N4—C18B	1.470 (16)	C7—H7B	0.9700
N4—C18A	1.48 (4)	C8—H8	0.9300
C1—C2	1.365 (10)	C10—H10	0.9300
C1—C6	1.365 (10)	C11—H11	0.9300
C2—C3	1.356 (11)	C13—H13	0.9300
C3—C4	1.359 (13)	C14—H14	0.9300
C4—C5	1.391 (11)	C15—H15A	0.9700
C5—C6	1.377 (10)	C15—H15B	0.9700
C6—C7	1.505 (8)	C17—H17	0.9300
C8—C9	1.477 (8)	C18A—H18B	0.9700
C9—C10	1.378 (8)	C18A—H18A	0.9700
C9—C14	1.378 (8)	C18B—H18D	0.9700
C10—C11	1.382 (8)	C18B—H18C	0.9700
C11—C12	1.375 (8)	C19A—H19A	0.9300
C12—C13	1.384 (7)	C19B—H19B	0.9300
C13—C14	1.381 (8)	C20A—H20B	0.9400
C15—C16	1.490 (8)	C20A—H20A	0.9300
C16—C17	1.342 (8)	C20B—H20D	0.9300
C18A—C19A	1.52 (5)	C20B—H20C	0.9300
C18B—C19B	1.50 (2)		
			
C12—O1—C15	117.3 (4)	C5—C4—H4	120.00
C7—N1—C8	115.9 (5)	C4—C5—H5	120.00
N3—N2—C16	107.8 (4)	C6—C5—H5	120.00
N2—N3—N4	106.8 (4)	N1—C7—H7A	110.00
N3—N4—C17	111.2 (4)	N1—C7—H7B	110.00
N3—N4—C18B	124.0 (9)	C6—C7—H7A	110.00
N3—N4—C18A	110.6 (17)	C6—C7—H7B	110.00
C17—N4—C18B	124.8 (9)	H7A—C7—H7B	108.00
C17—N4—C18A	138.1 (17)	N1—C8—H8	119.00
C2—C1—C6	121.4 (6)	C9—C8—H8	119.00
C1—C2—C3	119.6 (7)	C9—C10—H10	120.00
C2—C3—C4	120.7 (8)	C11—C10—H10	120.00
C3—C4—C5	119.8 (7)	C10—C11—H11	120.00
C4—C5—C6	119.6 (6)	C12—C11—H11	120.00
C1—C6—C5	118.9 (6)	C12—C13—H13	120.00
C1—C6—C7	120.7 (5)	C14—C13—H13	120.00
C5—C6—C7	120.4 (6)	C9—C14—H14	119.00
N1—C7—C6	109.9 (4)	C13—C14—H14	119.00
N1—C8—C9	122.8 (5)	O1—C15—H15A	110.00
C8—C9—C10	123.2 (5)	O1—C15—H15B	110.00
C8—C9—C14	118.3 (5)	C16—C15—H15A	110.00
C10—C9—C14	118.6 (5)	C16—C15—H15B	110.00
C9—C10—C11	120.4 (5)	H15A—C15—H15B	108.00
C10—C11—C12	120.8 (5)	N4—C17—H17	128.00
O1—C12—C11	115.6 (5)	C16—C17—H17	128.00
O1—C12—C13	125.1 (5)	H18A—C18A—H18B	109.00
C11—C12—C13	119.2 (5)	N4—C18A—H18B	111.00
C12—C13—C14	119.5 (5)	C19A—C18A—H18A	110.00
C9—C14—C13	121.5 (5)	N4—C18A—H18A	110.00
O1—C15—C16	109.1 (4)	C19A—C18A—H18B	110.00
N2—C16—C15	120.1 (5)	N4—C18B—H18D	109.00
N2—C16—C17	109.5 (5)	C19B—C18B—H18C	110.00
C15—C16—C17	130.4 (6)	N4—C18B—H18C	110.00
N4—C17—C16	104.8 (5)	H18C—C18B—H18D	108.00
N4—C18A—C19A	106 (3)	C19B—C18B—H18D	110.00
N4—C18B—C19B	110.6 (12)	C20A—C19A—H19A	125.00
C18A—C19A—C20A	111 (3)	C18A—C19A—H19A	124.00
C18B—C19B—C20B	120.9 (19)	C18B—C19B—H19B	120.00
C2—C1—H1	119.00	C20B—C19B—H19B	120.00
C6—C1—H1	119.00	C19A—C20A—H20A	121.00
C1—C2—H2	120.00	C19A—C20A—H20B	120.00
C3—C2—H2	120.00	H20A—C20A—H20B	120.00
C2—C3—H3	120.00	C19B—C20B—H20C	120.00
C4—C3—H3	120.00	C19B—C20B—H20D	120.00
C3—C4—H4	120.00	H20C—C20B—H20D	120.00
			
C12—O1—C15—C16	−178.3 (4)	C4—C5—C6—C1	−0.1 (10)
C15—O1—C12—C11	179.3 (5)	C1—C6—C7—N1	−70.5 (7)
C15—O1—C12—C13	0.2 (7)	C5—C6—C7—N1	106.7 (6)
C7—N1—C8—C9	−178.6 (5)	N1—C8—C9—C14	−172.7 (5)
C8—N1—C7—C6	112.8 (6)	N1—C8—C9—C10	8.0 (9)
C16—N2—N3—N4	0.8 (6)	C8—C9—C14—C13	−178.9 (5)
N3—N2—C16—C15	179.1 (5)	C8—C9—C10—C11	178.0 (5)
N3—N2—C16—C17	−0.2 (6)	C10—C9—C14—C13	0.3 (8)
N2—N3—N4—C18B	−178.1 (9)	C14—C9—C10—C11	−1.2 (8)
N2—N3—N4—C17	−1.2 (6)	C9—C10—C11—C12	1.5 (8)
C18B—N4—C17—C16	177.9 (9)	C10—C11—C12—C13	−0.8 (8)
N3—N4—C17—C16	1.1 (6)	C10—C11—C12—O1	−180.0 (5)
C17—N4—C18B—C19B	−99.3 (15)	C11—C12—C13—C14	−0.1 (8)
N3—N4—C18B—C19B	77.2 (16)	O1—C12—C13—C14	179.0 (5)
C2—C1—C6—C5	0.6 (10)	C12—C13—C14—C9	0.3 (8)
C2—C1—C6—C7	177.8 (6)	O1—C15—C16—N2	89.9 (6)
C6—C1—C2—C3	−1.3 (11)	O1—C15—C16—C17	−91.1 (7)
C1—C2—C3—C4	1.6 (12)	N2—C16—C17—N4	−0.5 (6)
C2—C3—C4—C5	−1.2 (12)	C15—C16—C17—N4	−179.7 (5)
C3—C4—C5—C6	0.4 (11)	N4—C18B—C19B—C20B	122 (2)
C4—C5—C6—C7	−177.4 (6)		

### Hydrogen-bond geometry (Å, º)

Cg2 is the centroid of the C1–C6 phenyl ring.

**Table:**

*D*—H···*A*	*D*—H	H···*A*	*D*···*A*	*D*—H···*A*
C17—H17···N3^i^	0.93	2.54	3.379 (7)	150
C18*B*—H18*D*···N1^ii^	0.97	2.52	3.42 (2)	153
C13—H13···*Cg*2^iii^	0.93	2.88	3.638 (6)	139
C18*B*—H18*C*···*Cg*2^iv^	0.97	2.95	3.706 (18)	135

Symmetry codes: (i) *x*, −*y*+3/2, *z*+1/2; (ii) −*x*+1, *y*+1/2, −*z*+1/2; (iii) −*x*, *y*+1/2, −*z*+1/2; (iv) −*x*+1, −*y*+1, −*z*.

## References

[bb1] Akkurt, M., Jarrahpour, A., Chermahini, M. M., Shiri, P. & Büyükgüngör, O. (2013*b*). *Acta Cryst.* E**69**, o1576. 10.1107/S1600536813025749 PMC3790434 24098253

[bb2] Akkurt, M., Jarrahpour, A., Chermahini, M. M., Shiri, P. & Tahir, M. N. (2013*a*). *Acta Cryst.* E**69**, o247. 10.1107/S1600536813000755 PMC3569780 23424526

[bb3] Calligaris, M. & Randaccio, L. (1987). *Comprehensive Coordination Chemistry*, Vol. 2, p. 715. Oxford: Pergamon.

[bb4] Dikusar, E. A. & Kozlov, N. G. (2006). *Russ. J. Org. Chem.* **42**, 369–375.

[bb5] Farrugia, L. J. (2012). *J. Appl. Cryst.* **45**, 849–854.

[bb6] Macho, V., Kralik, M., Hudec, J. & Cingelova, J. (2004). *J. Mol. Catal. A Chem.* **209**, 69–73.

[bb7] Sheldrick, G. M. (2008). *Acta Cryst.* A**64**, 112–122. 10.1107/S0108767307043930 18156677

[bb8] Stoe & Cie (2002). *X-AREA* and *X-RED32* Stoe & Cie, Darmstadt, Germany.

[bb9] Yap, A. H. & Weinreb, S. M. (2006). *Tetrahedron Lett.* **47**, 3035–3038.

[bb10] Yu, H.-X., Ma, J.-F., Xu, G.-H., Li, S.-L., Yang, J., Liu, Y.-Y. & Cheng, Y.-X. (2006). *J. Organomet. Chem.* **691**, 3531–3539.

